# Ideal nodal rings of one-dimensional photonic crystals in the visible region

**DOI:** 10.1038/s41377-022-00821-9

**Published:** 2022-05-12

**Authors:** Wei-Min Deng, Ze-Ming Chen, Meng-Yu Li, Chao-Heng Guo, Zhong-Tao Tian, Ke-Xin Sun, Xiao-Dong Chen, Wen-Jie Chen, Jian-Wen Dong

**Affiliations:** School of Physics & State Key Laboratory of Optoelectronic Materials and Technologies, Sun Yat-sen University, 510275 Guangzhou, China

**Keywords:** Optics and photonics, Optical physics

## Abstract

Three-dimensional (3D) artificial metacrystals host rich topological phases, such as Weyl points, nodal rings, and 3D photonic topological insulators. These topological states enable a wide range of applications, including 3D robust waveguides, one-way fiber, and negative refraction of the surface wave. However, these carefully designed metacrystals are usually very complex, hindering their extension to nanoscale photonic systems. Here, we theoretically proposed and experimentally realized an ideal nodal ring in the visible region using a simple 1D photonic crystal. The π-Berry phase around the ring is manifested by a 2π reflection phase’s winding and the resultant drumhead surface states. By breaking the inversion symmetry, the nodal ring can be gapped and the π-Berry phase would diffuse into a toroidal-shaped Berry flux, resulting in photonic ridge states (the 3D extension of quantum valley Hall states). Our results provide a simple and feasible platform for exploring 3D topological physics and its potential applications in nanophotonics.

## Introduction

In the study of topological states, topological semimetals^1–3^, featured by their symmetry-protected band degeneracies, serve as the parent states of various types of topological gapped states and have generated much research interest. These gapless band structures were usually studied in two- or three-dimensional (3D) lattice crystal, good examples are graphene^4,5^, and Dirac/Weyl semimetal^6,7^. Depending on the symmetries of the system, these stable degeneracies can occur at isolated points^6,7^, along closed lines^8–10^ or even on surfaces^11,12^ in 3D momentum space. Non-symmorphic symmetries and time-reversal symmetry can lead to nodal surfaces at the Brillouin zone boundary^13^. So far, nodal surfaces have been demonstrated in acoustic^14,15^ and photonic^16^ systems. In principle, for a system with higher symmetry, the topological band touching would occur in the higher-dimensional manifold. By lowering the crystal symmetries through certain types of interaction (spin-orbit interaction or external field), these degeneracies can be lifted, and the gapless states would transit to a rich variety of gapped topological states (such as topological insulating states or 3D quantum Hall states), along with the in-gap excitations guaranteed by the nontrivial band topology.

Apart from topological states of matter, topological band theory and the relevant concepts apply equally well to the photonic system^17–20^ and have recently inspired many novel applications^21–24^ in nanophotonics, such as backscattering-immune waveguides^25–29^, robust delay lines^30^, and high-performance lasers^31–34^. Most of these devices were based on 2D lattice crystals (e.g., photonic quantum spin/valley Hall systems) for their easier fabrication. But the 3D topological states and their topological effects on optical wavelength scale remain untamed, mostly because they are usually accompanied by complex 3D structures^35–42^. This may limit the development of topological photonics and its potential application in nanophotonics. Thus, it would be highly desirable if one could achieve a 3D topological state using a simple structure, preferably a 1D crystal^43^.

Here, we theoretically propose and experimentally realize an ideal nodal ring and the relevant topological gapped states in simple 1D photonic crystals (PCs). By taking off-axis momenta into account, we find that even a periodic layered medium can exhibit an ideal Dirac nodal ring dispersion without frequency variation in its 3D momentum space. By lowering the crystal’s symmetry, the ring degeneracies can transit to a rich variety of topological gapped states, such as photonic ridge state, the 3D extension of quantum valley Hall effect. These gapless/gapped bulk bands and the topological surface states are experimentally observed by angle-resolved reflection spectra in the visible region. Their nontrivial band topologies are analyzed through effective Hamiltonian and the calculated Berry curvature. Our results demonstrate a feasible platform for studying the 3D topological phases of light on the nanoscale and exploring their potential applications in nanophotonics.

## Results

**Fig. 1:**
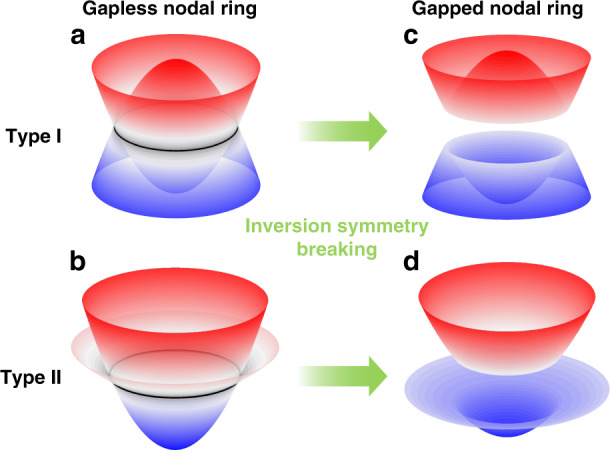
Schematics of type-I and type-II Dirac nodal rings. **a**, **b** Gapless nodal rings protected by inversion symmetry. **c**, **d** Gapped nodal rings after inversion symmetry breaking which exhibit ridge-like dispersion

A nodal ring can be deemed as the extrusion of 2D Dirac points along a closed-loop and thus it carries a quantized Berry phase of π. Its low-energy Hamiltonian takes the form of $H_N = \mu (k_r^2 - k_0^2)\sigma _z + v_zk_z\sigma _x + vk_r^2\sigma _0$, where *k*_0_ denotes the radius of the nodal ring and *σ*_*i*_ is Pauli matrix. On each cut plane containing *k*_*z*_ (e.g., the *k*_*y*_–*k*_*z*_ plane), the Hamiltonian reduces to a 2D Dirac Hamiltonian. According to the band tilting condition (the coefficients *v* and *μ*), nodal rings can be classified into type-I/II, as sketched in Fig. 1a, b. When |*v*/*μ*| > 1, the slopes of the two bands have the same signs, resulting in the tilted crossings along the nodal ring. Note that this tilting would not affect the topological characteristic of the nodal ring. These nodal line degeneracies, either type-I or type-II, are usually protected by PT symmetry. By breaking P or T symmetry (introducing a mass term *m*_*y*_(***k***)*σ*_*y*_ in *H*_*N*_), the nodal ring can break into multiple nodal points or be totally gapped, which depends on the specific form of the mass term *m*_*y*_ (***k***). For the simplest case that *m*_*y*_ is a constant, the nodal ring would be totally gapped and has a ridge-like dispersion, as sketched in Fig. 1c, d. These ridge states can be deemed as the 3D version of the quantum valley Hall effect.

In addition, these simplified models are isotropic along any radial direction, which is not the case for a real crystal. Even for a 3D crystal with discrete rotational symmetry, the nodal crossings are not guaranteed to occur at the same radius or the same eigenfrequency/energy. An ideal nodal ring can in principal exist in the systems with continuous rotational symmetry, for example, a layered medium or 1D PC. For a 1D layered medium, apart from the Bloch *k*_*z*_ in the out-of-plane direction, we take the in-plane components *k*_*x*_ and *k*_*y*_ into account. Then we can have a 3D momentum space for a 1D lattice structure and explore its 3D band topology.

**Fig. 2:**
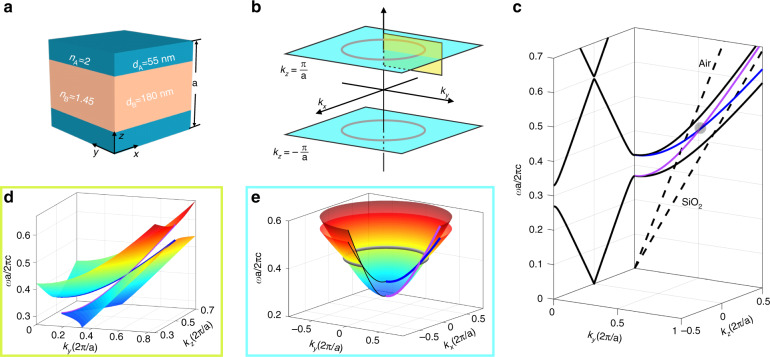
One-dimensional (1D) photonic crystal with ideal type-II nodal ring. **a** Unit cell of the 1D nodal ring photonic crystal (NRPC). It consists of three dielectric layers (A/B/A) and obeys inversion symmetry. The refractive index and thickness of layer A (B) is 2 (1.45) and 55 nm (180 nm), respectively. **b** 3D Brillouin zone of the 1D crystal. The two cyan planes highlight the Brillouin zone boundaries at *k*_*z*_ = ±*π*/*a*, on which the gray circles mark the nodal ring. **c** Band structures along *k*_*z*_ and *k*_*y*_ directions. The dashed lines represent the light lines of air and SiO_2_. **d** Zoom-in view of the tilted Dirac cone on the *k*_*y*_–*k*_*z*_ plane highlighted by yellow in **b**. **e** Band dispersion on the *k*_*x*_*–k*_*y*_ plane with *k*_*z*_ = π/a, which has a type-II nodal ring crossing (gray torus). For a better view of the nodal ring, only three quarters of the bands are shown

Consider a typical 1D PC with two dielectric components, as shown in Fig. 2a. Since the crystal has discrete translational symmetry in *z*-direction but continuous translational symmetry in *xy*-plane, the Brillouin zone of 1D PC is an infinite slab bounded by two planes of *k*_*z*_ = ±*π*/*a* (cyan planes in Fig. 2b). The left panel of Fig. 2c plots the *k*_*z*_-dispersion for normal propagation. A bandgap from 0.27(*c*/*a*) to 0.33(*c*/*a*) lies between the lowest two bands, which are doubly degenerate for s and p polarizations. As the in-plane component *k*_*y*_ increases (the right panel of Fig. 2c), the four bands split and curve upward with different velocities. The two p-polarized bands (blue and violet) linearly cross at *k*_*y*_ = 0.513(2*π*/*a*) and *ω* = 0.438(*c*/*a*). In fact, this is a type-II Dirac cone on the *k*_*y*_-*k*_*z*_ plane as illustrated in Fig. 2d. Since 1D PC is rotation-invariant, this tilted cone extrudes in an azimuthal direction (Fig. 2e), forming an ideal Dirac nodal ring without frequency variation. Note the nodal ring is formed by the p-polarized bands. In principle, s-polarized bands can also be employed to achieve nodal ring^43^ and more configurations of the nodal ring can be achieved if both polarizations are considered.

To confirm the band topology of the nodal ring, we derive its ***k***∙***p*** Hamiltonian via the transfer matrix method and it reads.

1 $$H = v_x\xi _{k_z}\sigma _x + (v_0\sigma _0 + v_z\sigma _z)\xi _{k_r}$$

where $\xi _{k_z} = (k_z - k_{z0})/k_{z0}$, $\xi _{k_r} = (k_r - k_{r_0})/k_{r_0}$. *k*_*z*0_ denotes the *k*_*z*_-plane the nodal ring lies on, $k_{r_0}$ is the radius of the nodal ring (see more details in Supplementary Information). On any *k*_*r*_–*k*_*z*_ cut plane, the effective Hamiltonian resembles a type-II Dirac Hamiltonian, implying the π Berry phase it carries^44^. Interestingly, the Dirac crossing in Fig. 2c happens to occur at the Brewster angle between the two dielectric materials^45^. But the Brewster effect is not a necessary condition for the existence of the nodal ring. Even for the 1D PCs without Brewster effect, such as ternary 1D PCs, the nodal ring still exists as long as the inversion symmetry is preserved (see Supplementary Fig. 2). Note here the polarization of the nodal ring degeneracy is different from that in ref. ^43^. In ref. ^43^, the authors focus on the realization of the nodal ring for both s and p polarizations. Their scheme, which has certain requirements on the optical paths for both layers, is applicable for binary 1D PCs. Here, we focus on the realization of the nodal ring only for p-polarization. Our scheme, which has no requirement on layer thickness, can be generalized to multicomponent 1D PCs.

**Fig. 3:**
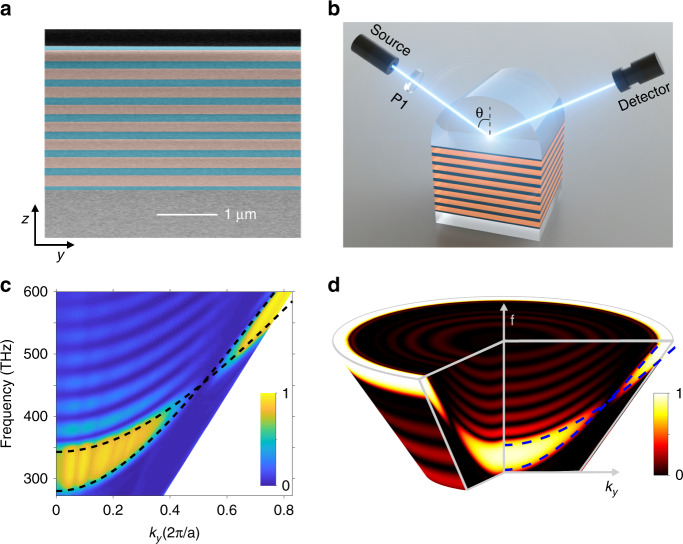
Experimental demonstration of the type-II nodal ring. **a** Scanning-electron-microscope (SEM) image of the fabricated sample. **b** Experimental setup for measuring the angle-resolved reflection spectra. The sample is contacted to a half cylindrical prism. P1, polarizer. **c**, **d** Measured (**c**) and calculated (**d**) angle-resolved reflection spectra for p-polarized incident light. The dashed curves represent the gap edges of the NRPC

For its simple 1D structure, such kind of nodal ring photonic crystals (NRPCs) can be readily fabricated using current nanofabrication techniques. Here, silicon nitride and silica are chosen to demonstrate our idea in the visible region. Figure 3a shows the scanning-electron-microscope (SEM) image of an 8-period NRPC fabricated using Chemical Vapor Deposition. In our experiment, the nodal ring degeneracies of bulk bands are investigated through angle-resolved reflection measurement, using the configuration depicted in Fig. 3b. Since the Dirac node lies below the light cone of air (Fig. 2c), the photonic states near the nodal ring cannot be directly excited by an incident beam from the air. Hence, a truncated hemi-cylindrical prism (JGS2 quartz glass, *n*_*p*_ = 1.45) is put on the upper sample to couple the incident beam into the NRPC. Figure 3d shows the calculated 3D reflection spectra for p-polarization as a function of *k*_*y*_ = *n*_*p*_*k*_0_sin*θ*_*i*_, where *θ*_*i*_ is the incident angle. As predicted by the bulk dispersion in Fig. 2c, the bandgap closes and reopens at *k*_*y*_ = 0.513(2*π*/*a*), corresponding to the position of the nodal point. Figure 3c shows the measured reflection spectra along *k*_*y*_ direction. One can see that there are two high-reflection regions, corresponding to two bandgaps connected by the nodal point. In contrast, the reflection is very low near the nodal point, due to the excitation of the bulk state at the nodal ring.

**Fig. 4:**
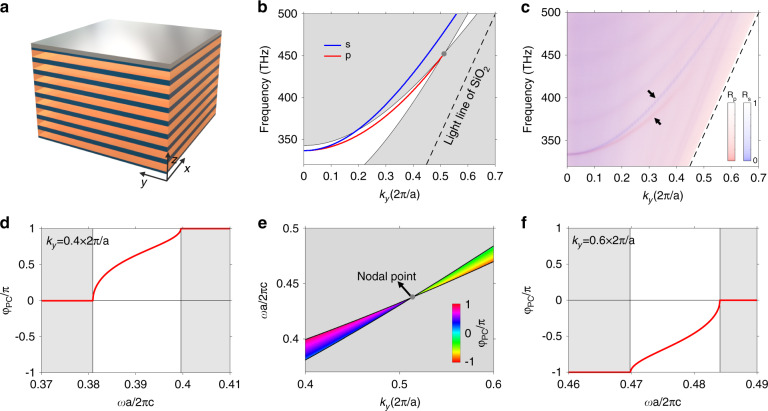
Drumhead surface states of the 1D gapless NRPC. **a** Schematic of the interface between 1D NRPC and silver. The silver film with a thickness of 40 nm is deposited on the 1D NRPC with 8 periods. **b** Projected band structure with s- (blue line) and p- (red line) polarized surface states. P-polarized surface states are pinned at the nodal point due to the phase winding around the nodal point. The black dashed line represents the light line of SiO_2_. **c** Overlaid plot of the measured angle-resolved reflection spectra for both polarizations. Two black arrows highlights two lines of reflection dips corresponding to the surface states. **e** Calculated p-polarized reflection phase (*φ*_PC_) of the NRPC. **d**, **f** Calculated *φ*_PC_ when *k*_*y*_ = 0.4 × 2*π*/*a* (**d**) and *k*_*y*_ = 0.6 × 2*π*/*a* (**f**). Note that in the vicinity of the Dirac nodal ring, there is a 2π phase winding around the nodal point which manifests its nontrivial Berry phase

The topological features of nodal rings are manifested in the surface properties when the NRPC is truncated in one dimension. Due to the nontrivial Berry phase around the nodal ring, the surface momentum space is divided into two regions (inside or outside the nodal ring) with π/0 Zak phases. For each (*k*_*x*_,*k*_*y*_), one can integrate a Zak phase along *k*_*z*_ direction (see more details in Supplementary Fig. S7). Numerical results show that the Zak phase inside (outside) the nodal ring is π (0), indicating the existence of a drumhead surface state pinned at the nodal ring. Experimentally, a 40 nm-thick silver film is deposited on the NRPC for 8 periods to study its surface, as depicted in Fig. 4a. The incident light can couple to the surface state via evanescent wave, as demonstrated by the simulation results in Supplementary Fig. 6. Similar to Fig. 3b, angle-resolved reflection measurements for both polarizations were conducted to obtain the reflection spectra of each polarization. Figure 4b plots the surface dispersion between NRPC and silver, where a p-polarized surface band (red line) is pinned at the nodal point (gray circle) and exhibits a drumhead dispersion inside the nodal ring. Besides, an s-polarized surface band exists and degenerates with the p-polarized band at *k*_*r*_ = 0 (for normal incidence). These surface bands can be clearly seen in our measured angle-resolved reflection data in Fig. 4c. In fact, there are always mid-gap surface states in the momentum space either inside or outside the nodal ring. Inside or outside the nodal ring depends on the surface truncation of NRPC (see more results in Supplementary Note 3). To gain a deep insight into the underlying mechanism, we calculate the reflection phase of NRPC in Fig. 4e. As an example, the reflection phases at *k*_*y*_ = 0.4(2*π*/*a*) and *k*_*y*_ = 0.6(2*π*/*a*) are plotted in Fig. 4d and Fig. 4f, respectively. One can see the reflection phase *φ*_PC_ exhibits a 2*π*-winding near the nodal point and the nodal point serves as a singularity of the reflection phase. Since a stable surface state should satisfy the condition of *φ*_PC_ + *φ*_Ag_ = 2*Nπ*, there would always be a surface band pinned at the nodal point, no matter the reflection phase of the other gapped material. In other words, the existence of surface states is protected by band topology (manifested as π Berry phase carried by the nodal ring). It should be noted that surface states between 1D PC and silver are also termed as Tamm optical states^46–51^. Here, we investigate the 2D Tamm optical states from the point of view of their topological protection. We show that the existence of surface states is actually the consequence of Berry phase carried by the nodal ring. Besides, the transverse spin feature of the surface state is also revealed (see more results in Supplementary Note 8). Applications of Tamm optical states have been investigated in many fields, such as nonlinear optics^52^, lasing^53,54^, quantum optics^55^, photonic devices^56^, hybrid systems^57,58^, and enhanced light-matter interaction^59,60^.

**Fig. 5:**
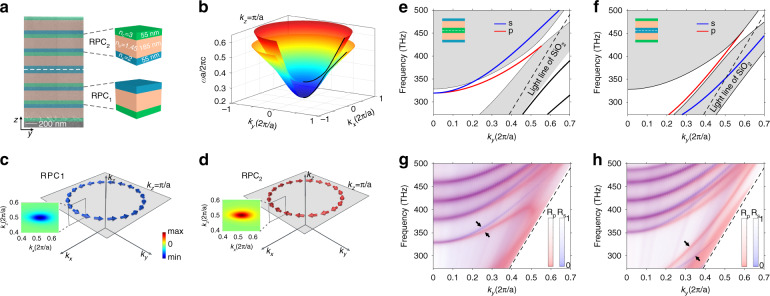
Gapped ridge state induced by inversion symmetry breaking. **a** Left panel: SEM image of the RPC_2_-RPC_1_ domain wall. The white dashed line highlights the boundary. Right panel: Two types of ridge photonic crystals (RPC_1_ and RPC_2_). **b** Bulk band structure of RPC_1_ on the plane of *k*_*z*_ = *π*/*a* with a partial gap opened in the vicinity of nodal ring. **c**, **d** Localized Berry vortexes for two RPCs. The insets show the cross-sectional views of the Berry curvature. Note that the Berry vortexes of two RPCs flow in opposite directions (clockwise or counter-clockwise) since they are linked by inversion. **e** Projected band of RPC_1_–RPC_2_ domain wall. Gapless surface states traverse the partial gap due to the opposite Berry vortexes of two RPCs. **g** Measured angle-resolved reflection spectra for both polarizations. Two lines of reflection dips (highlighted by the black arrows) inside the bandgap demonstrates the topological surface states. **f**, **h** Similar to (**e**) and (**g**), but for RPC_2_-RPC_1_ domain wall

In 2D photonic system, Dirac cones can be gapped by inversion symmetry breaking and resulting in the photonic valley Hall states^61–64^. As its 3D extrusion along the azimuthal angle, the nodal ring degeneracy can be lifted and deform into a ridge-like band structure (Fig. 1d). Hereafter we term the crystal with ridge dispersion as ridge photonic crystal (RPC). As shown in Fig. 5a, we break the inversion symmetry by replacing the bottom (top) layer A by a layer C with a refractive index of 3, named as RPC_1_ (RPC_2_). Figure 5b shows the bulk band structure of RPC_1_. One can see that the original nodal ring degeneracy is lifted, leading to a partial bandgap. Its effective Hamiltonian is written as $H_1 = \mu (k_r^2 - k_0^2)\sigma _z + v_zk_z\sigma _x + vk_r^2\sigma _0 + m\sigma _y$, where the symmetry breaking introduces a rotational-invariant mass term into *H*_1_. Meanwhile, the π Berry phase localized at the nodal ring would spread out into a toroidal-shaped Berry flux flowing in the counter-clockwise direction. Figure 5c shows the lower band’s Berry curvature of RPC_1_, whose inset plots a zoom-in view on the cut plane of *k*_*y*_ = 0. Since RPC_1_ and RPC_2_ are inversion partners of each other, they share an identical bulk band dispersion but have different eigen wave functions with opposite Berry curvatures. Numerical calculations in Fig. 5c, d show that the Berry vortexes of RPC_1_ and RPC_2_ are localized near the ridges and flow in opposite directions, indicating their distinct topological properties.

When two RPCs with distinct topological properties are joined together and form a domain wall, an in-gap surface state traversing the bandgap is expected. Figure 5e-h discusses two surface configurations, RPC_1_-RPC_2_ domain wall (Fig. 5e) and RPC_2_-RPC_1_ domain wall (Fig. 5f). Both calculated surface dispersions in Fig. 5e, f have a gapless p-polarized surface band (red line), which implies a conical-frustum-shaped dispersion near the ridge. It means that these topological surface states can propagate in all directions along with the interface between two RPCs (see more results in Supplementary Note 9). In experiment, we fabricate two domain wall samples with different configurations. The left panel in Fig. 5a shows the SEM image of the RPC_2_-RPC_1_ domain wall. The measured angle-resolved spectra in Fig. 5g, h show good agreement with the calculated dispersions and demonstrate the gapless surface states between two RPCs.

## Discussion

In conclusion, we propose and experimentally demonstrate an ideal type-II nodal ring using simple 1D structures. As the most remarkable feature of Dirac nodal ring, the drumhead surface states pinned at the ring are experimentally observed. By breaking inversion symmetry, the nodal ring state can transit to a photonic ridge state, whose nontrivial topology is signified by a clockwise/counter-clockwise Berry vortex in momentum space. The topological surface states between two RPCs with opposite Berry vortexes are also demonstrated. Compared to the complex 3D metamaterials, our 1D PCs are easier to design and fabricate, which facilitate the future application of 3D topological states in nanophotonics^65–68^, such as resonant scattering^69^ and negative refraction^70^. Besides, since the nodal ring can transform to Weyl point by symmetry breaking^71^, our work makes it possible to realize Weyl point and associated topological phenomena in 1D PCs.

## Materials and methods

### Numerical simulation

The band structures were calculated by MIT Photonic Bands (MPB)^72^, which is based on the plane-wave expansion (PWE) method. Reflection spectra were calculated by the transfer matrix method.

### Sample fabrication

Films are deposited on a quartz substrate by inductance coupled plasma-enhanced chemical vapor deposition system (ICP-CVD, Oxford Instruments PlasmaPro System 100). The deposition rate is 12 nm min^−1^ for SiO_2_, 13.5 nm min^−1^ for Si_x_N_y_, and 7 nm min^−1^ for silicon-rich nitride. The Ag film is deposited by electron beam evaporation (DE400DHL, DE Technology Inc.) and the corresponding deposition rate is 6 nm min^−1^.

### Optical measurements

The refractive index and extinction coefficient of silicon-rich nitride were determined from spectrometry ellipsometry measurements (Sentech SE400). Angle-resolved reflection spectra of films were measured by an angle-resolved spectrum system (R1-UV, Ideaoptics, China). A truncated hemi-cylindrical prism (*n*_*p*_ = 1.45, radius R = 8 mm) is put on the sample to couple the incident beam into the sample. In order to avoid the influence of reflected light from the interface between the substrate and air, the bottom surface of the substrate is contacted to an extra-thick glass substrate (25 × 25 × 20 mm) by means of index-matching liquid. In the experiment, the sample size in the x–y plane (20 × 20 mm) is much larger than the spot size of the incident beam (*ω*_0_ ≈ 1 mm), indicating the edge effect of the sample can be neglected. Besides, the finite size of the incident beam would not affect the observation of the nodal ring, either. Considering the focusing effect of the cylindrical lens, the lateral wavevector induced by the beam divergence is estimated as ∆*k*_*y*_ ≈ 2.80 × 10^5^m^−1^ (see more details in Supplementary Note 10), which is much smaller than the *k*_*y*_ value at the nodal ring (*k*_*y*__0_ = 0.513 × 2*π*/*a* ≈ 1.11 × 10^7^m^−1^). So, the divergence of the incident beam does not affect the observation of the nodal ring in the experiment.

## Supplementary information

Supplementary Information

## Data Availability

The authors declare that all data supporting the findings of this study are available within the paper and its Supplementary Information files
