# Supplementary material for: Ideal nodal rings of one-dimensional photonic crystals in the visible region
Source: Light Sci Appl. 2022 May 12;11:134. doi: 10.1038/s41377-022-00821-9 (PMC9098453; doi:10.1038/s41377-022-00821-9)
Supplement: Supplementary file 1 — Supplementary Information [file 41377_2022_821_MOESM1_ESM.docx]

Supplementary Information for

**“Ideal Nodal Rings of One-Dimensional Photonic Crystals in the Visible Region”**

Wei-Min Deng, Ze-Min Chen, Meng-Yu Li, Chao-Heng Guo, Zhong-Tao Tian, Ke-Xin Sun, Xiao-Dong Chen, Wen-Jie Chen^*^, and Jian-Wen Dong^*^

School of Physics & State Key Laboratory of Optoelectronic Materials and Technologies, Sun Yat-sen University, Guangzhou 510275, China.

^*^Corresponding author: chenwenj5@mail.sysu.edu.cn; dongjwen@mail.sysu.edu.cn

## 1. Effective Hamiltonian near the nodal ring degeneracies


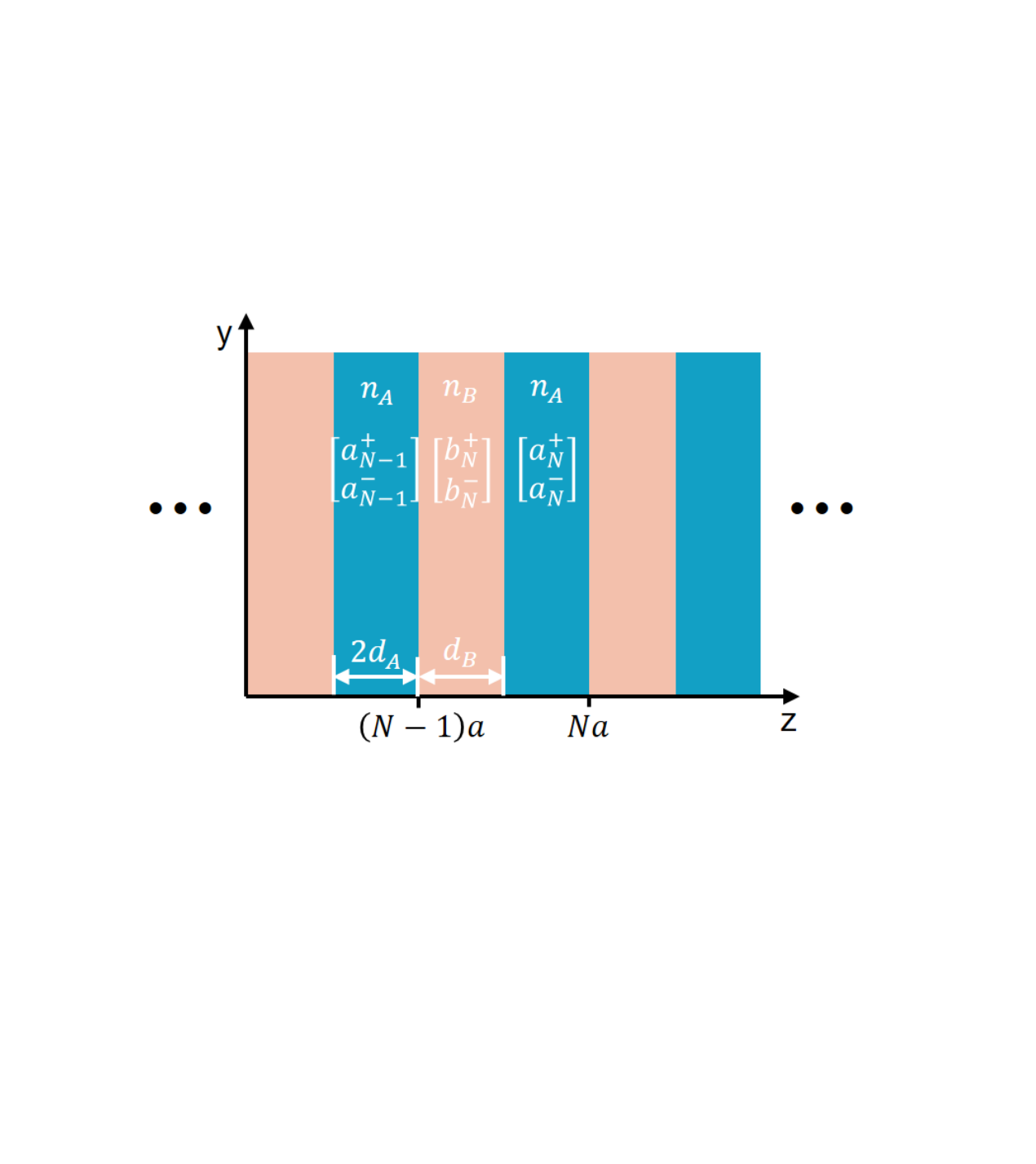


Fig. S1 Schematic of a one-dimensional photonic crystal.

In this section, we derive the effective Hamiltonian of the one-dimensional (1D) photonic crystal (PC) when the band dispersion near the nodal ring is considered. At first, we derive the wave equation of p-polarized wave according to the transfer matrix and Bloch boundary condition. The transfer matrix ***T*** of the 1D PC in Fig. S1 can be expressed as^1^

$$T=\left(\begin{matrix} T_{11} & T_{12} \\ T_{21} & T_{22} \end{matrix}\right)$$

where

$$T_{11}=e^{-ik_{zA}2d_{A}}\left[\cos k_{zB}d_{B}-\frac{i}{2}\left(\frac{k_{zB}n_{A}^{2}}{k_{zA}n_{B}^{2}}+\frac{k_{zA}n_{B}^{2}}{k_{zB}n_{A}^{2}}\right)\sin k_{zB}d_{B}\right]$$

$$T_{12}=e^{ik_{zA}2d_{A}}\left[-\frac{i}{2}\left(\frac{k_{zB}n_{A}^{2}}{k_{zA}n_{B}^{2}}-\frac{k_{zA}n_{B}^{2}}{k_{zB}n_{A}^{2}}\right)\sin k_{zB}d_{B}\right]$$

$$T_{21}=e^{-ik_{zA}2d_{A}}\left[\frac{i}{2}\left(\frac{k_{zB}n_{A}^{2}}{k_{zA}n_{B}^{2}}-\frac{k_{zA}n_{B}^{2}}{k_{zB}n_{A}^{2}}\right)\sin k_{zB}d_{B}\right]$$

$$T_{22}=e^{ik_{zA}2d_{A}}\left[\cos k_{zB}d_{B}+\frac{i}{2}\left(\frac{k_{zB}n_{A}^{2}}{k_{zA}n_{B}^{2}}+\frac{k_{zA}n_{B}^{2}}{k_{zB}n_{A}^{2}}\right)\sin k_{zB}d_{B}\right]$$

In Eq. - Eq. , $k_{zA}=\sqrt{\left( n_{A}\omega/c \right)^{2}-k_{y}^{2}}$ and $k_{zB}=\sqrt{\left( n_{B}\omega/c \right)^{2}-k_{y}^{2}}$. In the $N^{th}$ period of 1D PC, If we denote the amplitudes of forward-propagating and backward-propagating waves in the layer A as $a_{N}^{+}$ and $a_{N}^{-}$, respectively, we have

$$\left[\begin{matrix} a_{N-1}^{+} \\ a_{N-1}^{-} \end{matrix}\right]=T(\omega,k_{y})\left[\begin{matrix} a_{N}^{+} \\ a_{N}^{-} \end{matrix}\right]$$

According to the Bloch boundary condition, we have

$$\left[\begin{matrix} a_{N-1}^{+} \\ a_{N-1}^{-} \end{matrix}\right]=e^{-ik_{z}a}\left[\begin{matrix} a_{N}^{+} \\ a_{N}^{-} \end{matrix}\right]$$

Based on Eq. and Eq. , we can obtain the wave equation

$$[T(\omega,k_{y})-e^{-ik_{z}a}]\left[\begin{matrix} a_{N}^{+} \\ a_{N}^{-} \end{matrix}\right]=0$$

Then, we start to derive the effective Hamiltonian. First of all, we define three dimensionless parameters: $\xi_{k_{z}}=(k_{z}-k_{z0})/k_{z0}$, $\xi_{k_{y}}=(k_{y}-k_{y0})/k_{y0}$, and $\xi_{\omega}=(\omega-\omega_{0})/\omega_{0}$,, where $k_{y0}$ and $k_{z0}$ are the $k_{y}$ and $k_{z}$ values of the nodal point in the $k_{x}=0$ plane, respectively. $\omega_{0}$ is the frequency of the nodal ring. Note that the nodal ring exists in the $k_{z}=\pi/a$ plane. According to Eq. , we can know that $\boldsymbol{T}\left( \omega_{0},k_{y0} \right)=e^{-i\pi}=-1$. Near the nodal point, we expand $\boldsymbol{T}(\omega,k_{y})$ up to the first-order approximation and neglect other higher order terms. Then, the approximate transfer matrix can be expressed as

$$T(\omega,k_{y})\approx\left(\begin{matrix} -1+ic_{1}\xi_{k_{y}}+ic_{2}\xi_{\omega} & d_{1}\xi_{k_{y}}+d_{2}\xi_{\omega} \\ d_{1}^{*}\xi_{k_{y}}+d_{2}^{*}\xi_{\omega} & -1-(ic_{1}\xi_{k_{y}}+ic_{2}\xi_{\omega}) \end{matrix}\right)$$

where $c_{1}\text{=}\frac{k_{y0}^{2}e^{-2id_{A}k_{zA0}}}{2k_{zA0}^{2}k_{zB0}^{2}n_{A}^{2}n_{B}^{2}}(k_{zA0}(d_{B}k_{zB0}^{2}n_{A}^{4}+4d_{A}k_{zB0}^{2}n_{A}^{2}n_{B}^{2}+d_{B}k_{zA0}^{2}n_{B}^{4})cos(d_{B}k_{zB0})-k_{zB0}^{\text{1/2}}$

$(k_{zB0}^{\text{1/2}}(-k_{zA0}+2id_{A}k_{zA0}^{2}+k_{zA0}^{5/2})n_{A}^{4}+2id_{B}k_{zA0}^{2}k_{zB0}^{\text{1/2}}n_{A}^{2}n_{B}^{2}+k_{zA0}(k_{zA0}^{2}+(-1+2id_{A}k_{zA0})k_{zB0}^{\text{1/2}})n_{B}^{4})$.$sin(d_{B}k_{zB0}))$, $c_{2}=-\frac{\omega_{0}^{2}e^{-2id_{A}k_{zA0}}}{2c^{2}k_{zA0}^{2}k_{zB0}^{2}n_{A}^{2}n_{B}^{2}}(k_{zA0}n_{B}^{2}((4d_{A}+d_{B})k_{zB0}^{2}n_{A}^{4}+d_{B}k_{zA0}^{2}n_{B}^{4})cos(d_{B}k_{zB0})$

$+k_{zB0}^{\text{1/2}}((-2id_{A}-k_{zA0}^{\text{1/2}})k_{zB0}^{5/2}n_{A}^{6}+k_{zA0}k_{zB0}^{\text{1/2}}n_{A}^{4}n_{B}^{2}+k_{zA0}(1-2i(d_{A}+d_{B})k_{zA0})k_{zB0}^{\text{1/2}}n_{A}^{2}n_{B}^{4}-k_{zA0}^{3}n_{B}^{6})$ $sin(d_{B}k_{zB0}))$,$d_{1}=-\frac{k_{y0}^{2}e^{2id_{A}k_{zA0}}}{2k_{zA0}^{2}k_{zB0}^{2}n_{A}^{2}n_{B}^{2}}(d_{B}k_{zA0}(-k_{zB0}^{2}n_{A}^{4}+k_{zA0}^{2}n_{B}^{4})cos(d_{B}k_{zB0})+k_{zB0}^{\text{1/2}}(k_{zB0}^{\text{1/2}}$

$(-k_{zA0}+(-2id_{A}+k_{zA0}^{\text{1/2}})k_{zB0}^{2})n_{A}^{4}+k_{zA0}(-k_{zA0}^{2}+(1+2id_{A}k_{zA0})k_{zB0}^{\text{1/2}})n_{B}^{4})sin(d_{B}k_{zB0}))$, $d_{2}=\frac{\omega_{0}^{2}e^{2id_{A}k_{zA0}}}{2c^{2}k_{zA0}^{2}k_{zB0}^{2}n_{A}^{2}n_{B}^{2}}(d_{B}k_{zA0}n_{B}^{2}(-k_{zB0}^{2}n_{A}^{4}+k_{zA0}^{2}n_{B}^{4})cos(d_{B}k_{zB0})+k_{zB0}^{\text{1/2}}((-2id_{A}+k_{zA0}^{\text{1/2}})k_{zB0}^{5/2}n_{A}^{4}$ $-k_{zA0}k_{zB0}^{\text{1/2}}n_{A}^{4}n_{B}^{2}+k_{zA0}(1+2id_{A}k_{zA0})k_{zB0}^{\text{1/2}}n_{A}^{2}n_{B}^{4}-k_{zA0}^{3}n_{B}^{6})sin(d_{B}k_{zB0}))$.Besides, we also exp-and the phase factor in Eq. near the nodal point and we obtain

$$e^{-ik_{z}a}\approx-1+ia(k_{z}-k_{z0})=-1+ic_{3}\xi_{k_{z}}$$

where $c_{3}=\pi$. Based on Eq. and Eq. ，the wave equation can be expressed as

$$\left(\begin{matrix} ic_{1}\xi_{k_{y}}+ic_{2}\xi_{\omega}-ic_{3}\xi_{k_{z}} & d_{1}\xi_{k_{y}}+d_{2}\xi_{\omega} \\ d_{1}^{*}\xi_{k_{y}}+d_{2}^{*}\xi_{\omega} & -(ic_{1}\xi_{k_{y}}+ic_{2}\xi_{\omega})-ic_{3}\xi_{k_{z}} \end{matrix}\right)\left(\begin{matrix} a_{N}^{+} \\ a_{N}^{-} \end{matrix}\right)=0$$

We then transform Eq. into the following form

$$M\left(\begin{matrix} -ia_{N}^{+} \\ ia_{N}^{-} \end{matrix}\right)=\xi_{\omega}\left(\begin{matrix} c_{2} & id_{2} \\ -id_{2}^{*} & c_{2} \end{matrix}\right)\left(\begin{matrix} -ia_{N}^{+} \\ ia_{N}^{-} \end{matrix}\right)$$

where $M=[-(c_{1}\xi_{k_{y}}-c_{3}\xi_{k_{z}}),\text{ }-id_{1}\xi_{k_{y}};\text{ }id_{1}^{*}\xi_{k_{y}},\text{ }-(c_{1}\xi_{k_{y}}+c_{3}\xi_{k_{z}})]$. The matrix on the right-hand side of Eq. can be further decomposed as

$$\left(\begin{matrix} c_{2} & id_{2} \\ -id_{2}^{*} & c_{2} \end{matrix}\right)=UVU^{\dagger}$$

where $U=[-i{d_{2}}/|d_{2}|,\text{ }i{d_{2}}/|d_{2}|;\text{ }1,\text{ }1]/\sqrt{2}$, $\left. V=[c_{2}-|d_{2}|,\text{ }0;\text{ }0,\text{ }c_{2}+|d_{2}| \right]$. After some formula simplification, Eq. can be expressed as the following eigenvalue equation

$V^{\frac{1}{2}}U^{\dagger}MUV^{-\frac{1}{2}}\left( \begin{matrix} -i\tilde{a}_{N}^{+} \\ i\tilde{a}_{N}^{-} \end{matrix} \right)=\xi_{\omega}\left( \begin{matrix} -i\tilde{a}_{N}^{+} \\ i\tilde{a}_{N}^{-} \end{matrix} \right)$

where $[-i\tilde{a}_{N}^{+}\text{ };\text{ }i\tilde{a}_{N}^{-}]=V^{\frac{1}{2}}U^{\dagger}[-ia_{N}^{+}\text{ };\text{ }ia_{N}^{-}]$. Therefore, we obtain the effective Hamiltonian in the 2D *k_y_*-*k_z_* plane

$$H_{1}=V^{-\frac{1}{2}}U^{\dagger}MUV^{-\frac{1}{2}}\text{=}v_{x}\xi_{k_{z}}\sigma_{x}+(v_{0}\sigma_{0}+v_{y}\sigma_{y}+v_{z}\sigma_{z})\xi_{k_{y}}$$

where $\sigma_{0}$ is the unitary matrix and $\sigma_{x,y,z}$ are Pauli matrixes. By applying the unitary transformation, the effective Hamiltonian can be expressed in a more compact form

$H_{2}=\tilde{U}^{-1}H\tilde{U}=v_{x}\xi_{k_{z}}\sigma_{x}+(v_{0}\sigma_{0}+\tilde{v}_{z}\sigma_{z})\xi_{k_{y}}$

where $\left. \tilde{U}=[cos(\theta\text{/2)},\text{ }i\sin(\theta\text{/2)};\text{ }i\sin(\theta\text{/2)},\text{ }cos(\theta\text{/2)} \right]$ and $\tan\theta=v_{y}/v_{z}$. Due to the continuous rotational symmetry in x-y plane, the effective Hamiltonian in 3D momentum space can be expressed as

$$H=v_{x}\xi_{k_{z}}\sigma_{x}+(v_{0}\sigma_{0}+v_{z}\sigma_{z})\xi_{k_{r}}$$

where $\xi_{k_{r}}$ is nodal ring’s momentum along radial direction in $k_{z}=\pi/a$ plane.

## 2. Nodal ring degeneracies in the absence of Brewster effect

In this section, we will demonstrate the nodal ring can still exist in the absence of Brewster effect, as long as the inversion symmetry is preserved. In the main text, we mention that for the 1D NRPC in Fig. S2a, the nodal ring crossing happens to occur at the Brewster angle. In fact, this kind of ring degeneracies ubiquitously exist in a binary 1D PC due to Brewster effect. A binary 1D PC is composed of a series of interfaces between A and B materials, as shown in Fig. S2a. Once the Brewster condition $\omega=ck_{y}\sqrt{\frac{1}{\left( n_{A} \right)^{2}}+\frac{1}{\left( n_{B} \right)^{2}}}$ is satisfied, all the A-B interfaces would be transparent for p-polarized wave. Then the p-polarized band gap would naturally close and forms a nodal ring at Brewster angle, see Fig. S2b & c.

However, this is not always the case when the 1D PC is composed of more than two materials. Fig. S2d takes a ternary 1D PC for example, which is composed of three materials A, B and D. In this case, there are two kinds of interfaces in this 1D photonic crystal, A-D interface and B-D interface. The Brewster line for A-D interface is written as $\omega=ck_{y}\sqrt{\frac{1}{\left( n_{A} \right)^{2}}+\frac{1}{\left( n_{D} \right)^{2}}}$ while the one for B-D interface is $\omega=ck_{y}\sqrt{\frac{1}{\left( n_{B} \right)^{2}}+\frac{1}{\left( n_{D} \right)^{2}}}$. Since $n_{A}\neq n_{B}$, these two Brewster lines do not overlap and the Brewster condition cannot be simultaneously fulfilled at these two interfaces. Generally speaking, the p-polarized degeneracies would be lifted and open a band gap, good examples are RPC_1_ and RPC_2_ in the main text. But for the ternary 1D PC in Fig. S2d inversion symmetry and time-reversal symmetry are preserved. This PT symmetry, together with the π-Berry phase near nodal ring, protects the ring degeneracies. From the calculated band structure along in Fig. S2e, one can see that band crossing along $k_{y}$ direction still exists. The two bands also linearly cross in $k_{z}$ direction (Fig. S2f), indicating that the band crossings in the $k_{x}-k_{y}$ plane indeed form a nodal ring. These results indicate Brewster effect is not a necessary condition for the existence of nodal ring in 1D PCs.


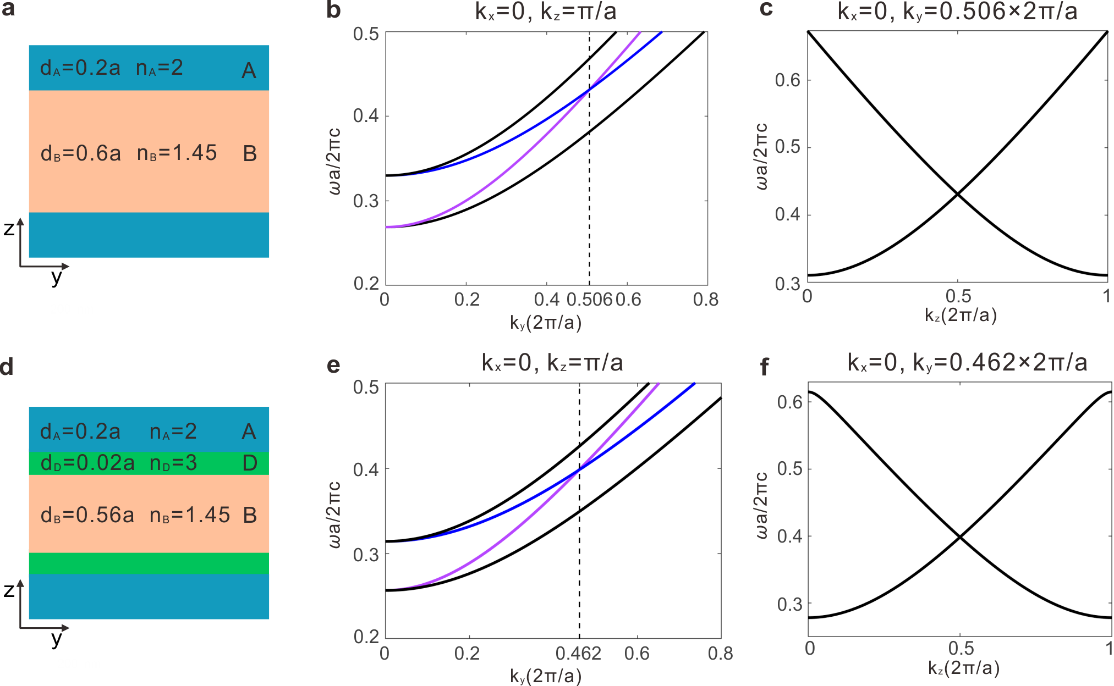


Fig. S2 (a) Unit cell of a binary 1D PC. (b) Bulk band along $k_{y}$ direction, keeping $k_{x}=0$ and $k_{z}=\pi/a$. (c) Bulk band along $k_{z}$ direction. (d) - (f): Similar to (a) - (c), but for a ternary 1D PC with inversion symmetry.

## 3. Existence of the drumhead surface state for different types of surface truncation

In this section, we will demonstrate the existence of the drumhead surface state does not depend on the surface truncation or the gapped material that cladding on the surface of PC. We consider three different types of surface truncation. The first column in Fig. S3 shows the schematic of these three truncations. The truncation1 in Fig. S3a is the case we considered in Fig. 4 of the main text. The second column in Fig. S3 shows the reflection phase of each truncation. As an example, the reflection phases at $k_{y}=0.4\times2\pi/a$ and $k_{y}=0.6\times2\pi/a$ are plotted in the third and fourth columns in Fig. S3. For truncation1, one can see that the $\varphi_{\mathrm{PC}}$ at $k_{y}=0.4\times2\pi/a$ together with the $\varphi_{\mathrm{PC}}$ at $k_{y}=0.6\times2\pi/a$ can cover the phase range from $-\pi$ to $\pi$. If the caldding gapped material has an reflection phase $\varphi_{1}$, the condition $\varphi_{\mathrm{PC}}+\varphi_{1}=0$ can always be satisfied. Thus, there is always a surface state resides in one of these two gaps. Similarly, by analyzing the reflection phases of truncation2 and truncation3, we can get the same conclusion.


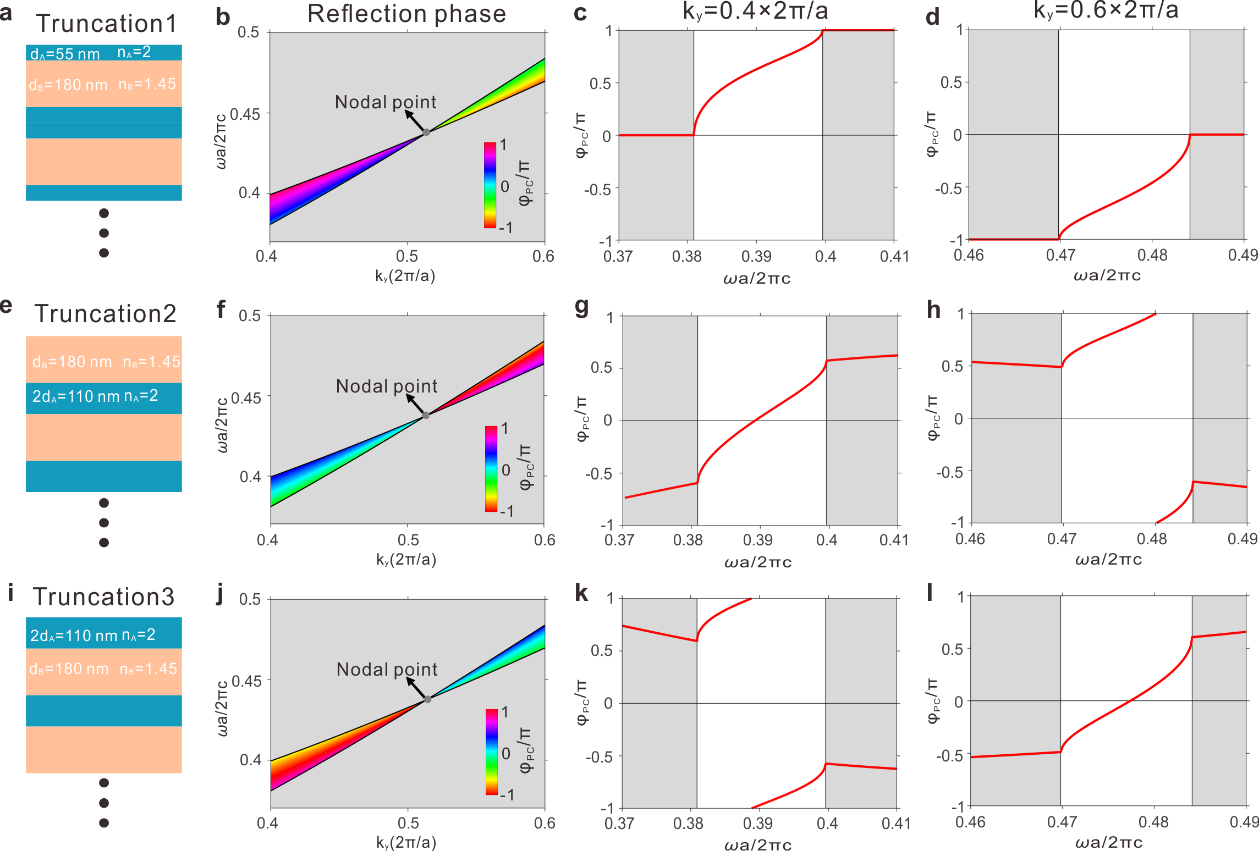


Fig. S3 Reflection phase for three types of surface truncation. (a) Schematic of truncation1. (b) Calculated p-polarized reflection phase ($\varphi_{\mathrm{PC}}$). (c), (d) Calculated $\varphi_{\mathrm{PC}}$ when $k_{y}=0.4\times2\pi/a$ (c) and $k_{y}=0.6\times2\pi/a$ (d). (e)-(h) Similar to (a)-(d), but for truncation2. (i)-(l), Similar to (a)-(d), but for truncation3.

## 4. Optical constants of silicon-rich nitride


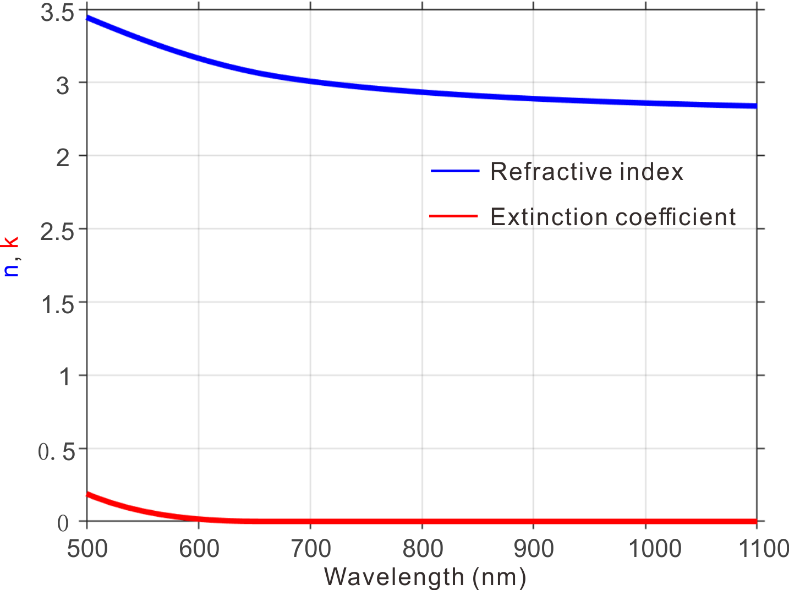


Fig. S4 Optical constants of silicon-rich nitride. Blue line: refractive index *n*; Red line: extinction coefficient *k*.

## 5. Measured and calculated reflection spectra of NRPC


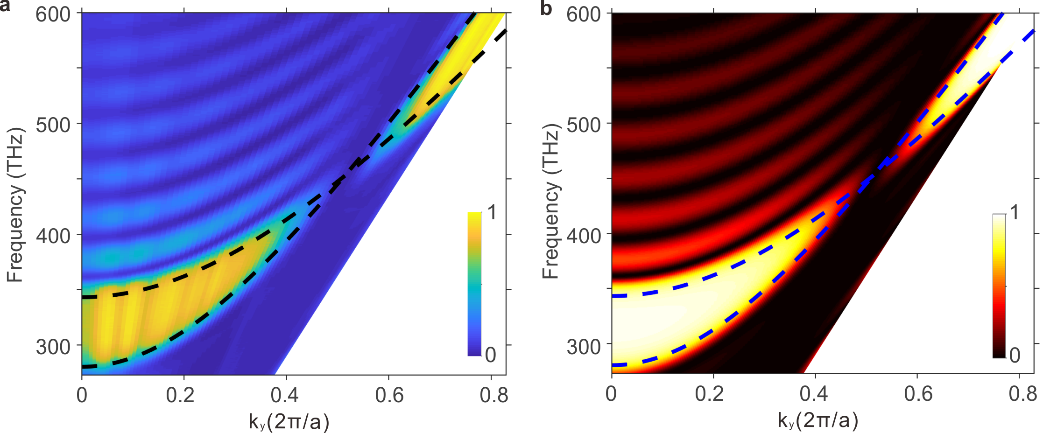


Fig. S5 (a) Measured and (b) calculated angle-resolved reflection spectra for p-polarized incident light. The dashed curves represent the gap edges of the NRPC.

## 6. Excitation of drumhead surface state by plane waves with different incident angles


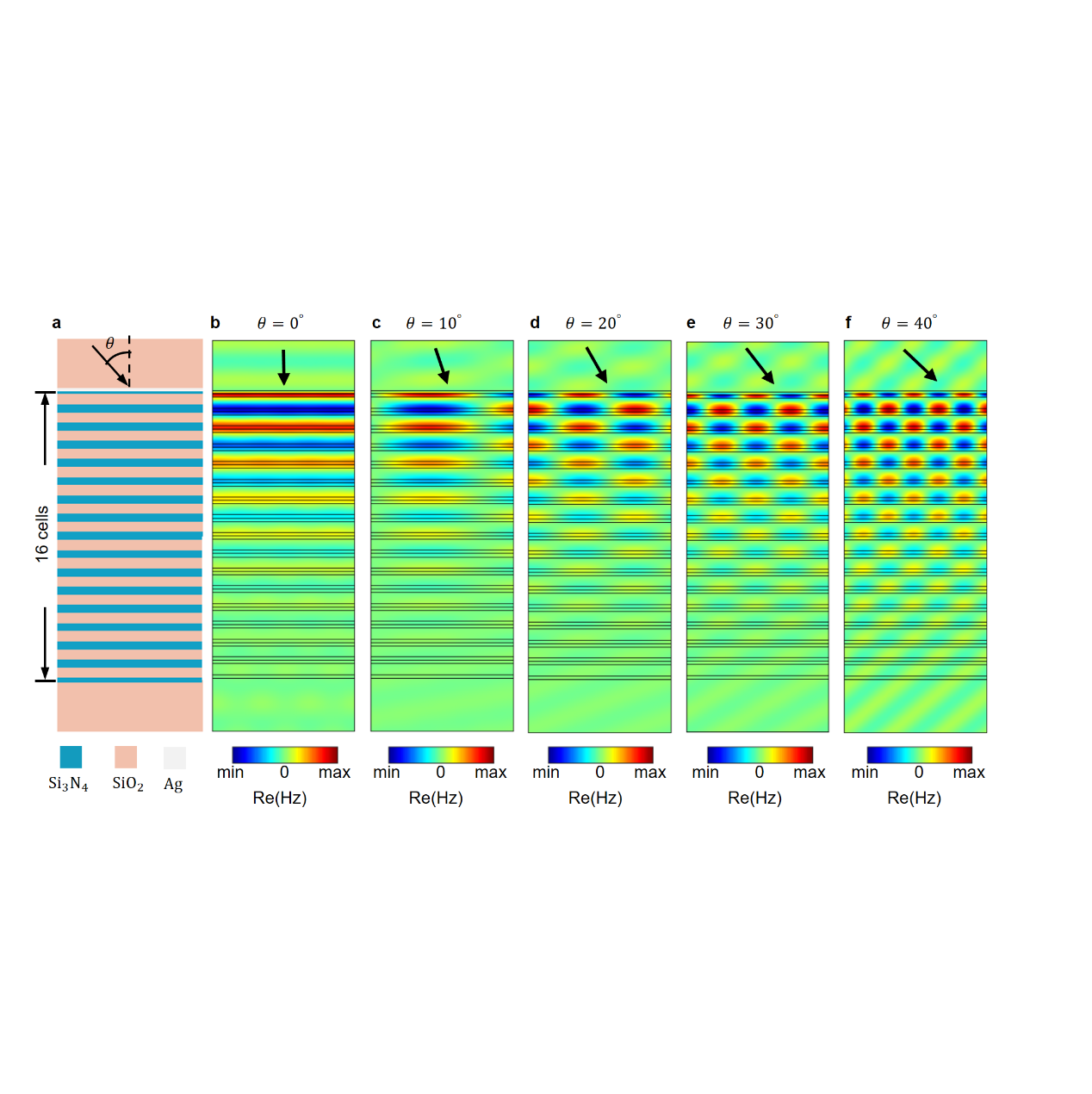


Fig. S6 (a) Schematic of the surface structure. (b) - (f): Excitation of drumhead surface state by plane waves for five different incident angles.

## 7. Zak phase distribution in *k_x_ - k_y_* plane

For each point in *k_x_ - k_y_* plane, the nodal ring photonic crystal can be deemed as a one-dimensional system along *k_z_* direction. The band topology of such 1D subsystem can be described by Zak phase. Due to the nontrivial Berry phase around the nodal ring, the *k_x_ - k_y_* plane is divided into two regions (inside or outside the nodal ring) with different Zak phases. To illustrate the Zak phase of each region, we choose one path inside the ring ($k_{x}=0, k_{y}=0.4\times2\pi/a$) and another path out side the ring ($k_{x}=0, k_{y}=0.6\times2\pi/a$) for comparison. These two paths are highlighted by red and blue lines in Fig. S7a. The p-polarized bulk bands along these two paths are shown in Fig. S7b and Fig. S7c, where the Zak phases are labeled in green while the band numbers are in red. The Zak phase of the 1^st^ band in Fig. S7b and Fig. S7c is calculated to be π and 0, respectively. Therefore, the Zak phase inside (outside) the nodal ring is π (0), as shown in Fig. S7d. And the nodal ring is the phase transition boundary between these two phases, where band inversion occurs. Similar to Fig. S7b and Fig. S7c, Fig. S7e and Fig. S7f shows the s-polarized bulk bands and corresponding Zak phases.


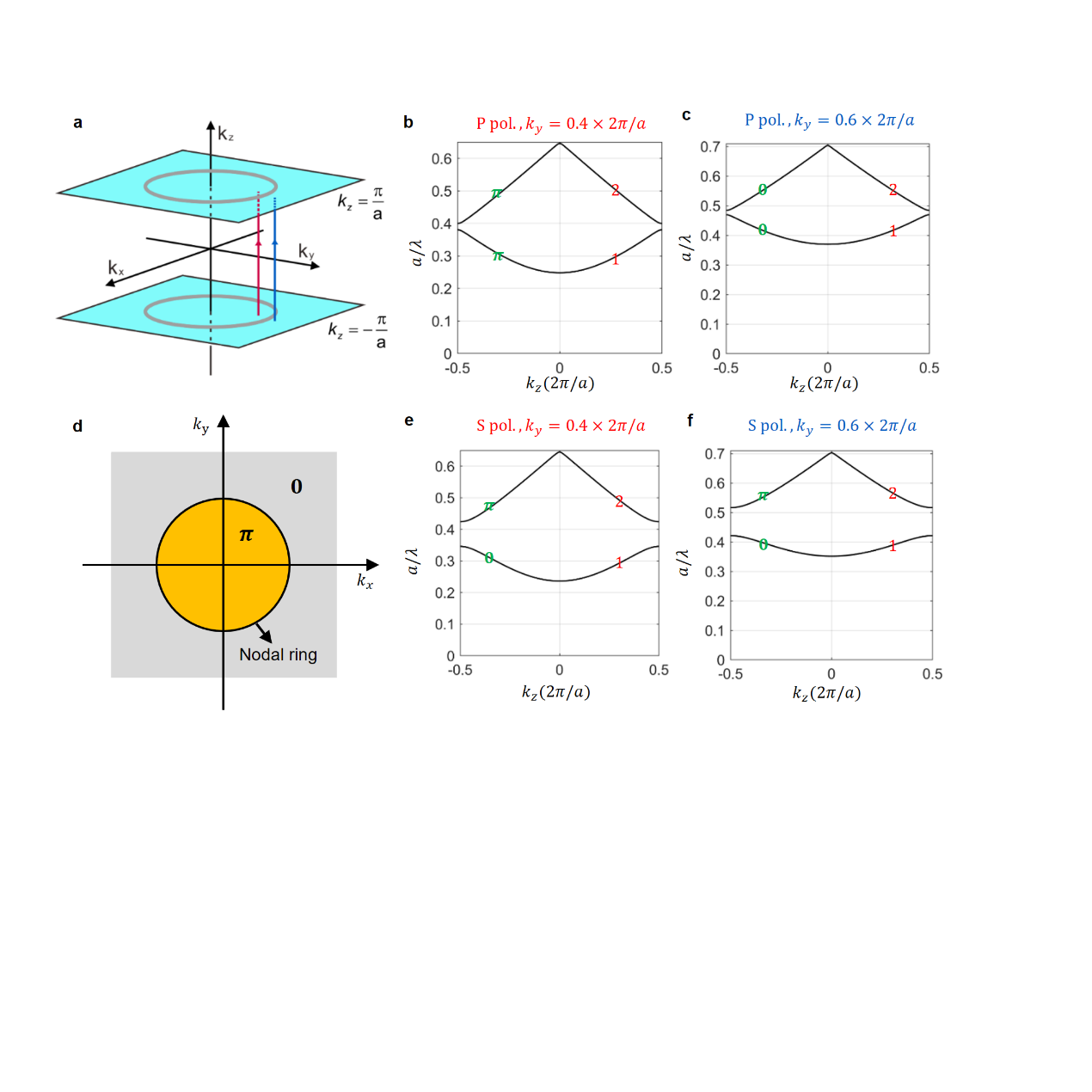


Fig. S7 (a) Brillouin zone and the integration path for Zak phase calculation. (b) and (c): P-polarized bulk band along $k_{z}$ while keeping (b) $k_{y}=0.4\times2\pi/a$ and (c) $k_{y}=0.6\times2\pi/a$. (d) Zak phase distribution of 1^st^ p-polarized band in surface k space. (e) and (f): Similar to (b) and (c), but for s-polarization. The Zak phases are labeled in green while the band numbers in red.

## 8. Transverse spin of the drumhead surface state

Many studies about photonic topological edge states reveal that topological edge states may exhibit transverse spin feature^2-4^, where the eigen fields are in-plane circularly polarized. In some cases, the transverse spin of topological edge state is locked to the propagation direction of the edge state, which enables the unidirectional excitation of the edge state. Here, we find the drumhead surface states also possess transverse spin. Fig. S8c calculates the normalized Stokes polarization parameter S_3_ near the interface for p-polarized surface state at $k_{y}=0.3\times2\pi/a$. Stokes polarization parameter S_3_ describes the degree of circular polarization and it is defined as^5^ ${S_{3}(z)=i(E_{x}(z)E_{y}^{*}(z)-E_{y}(z)E_{x}^{*}(z))}/{({|E_{x}(z)|}^{2}+{|E_{y}(z)|}^{2})}$, where $E_{x}(z)$ and $E_{y}(z)$ are the electric field components of the surface state. Note the distributions of $E_{x}$ and $E_{y}$ are non-uniform, thus the value of *S_3_* is position-dependent. *S_3_* = 1 and -1 represents right-handed circular polarization (RCP) and LCP, respectively. From Fig. S8c, one can see that the value of *S_3_* is close to 1 near the edge of Si_3_N_4_ layer, exhibiting transverse spin feature of the surface state. If a RCP (LCP) source is placed near the edge of Si_3_N_4_ layer, most of the energy will couple to the rightward (leftward) edge state, as demonstrated by the simulation result in Fig. S8d. This property may have application in manipulating chiral emission of the quantum emitter^6^.


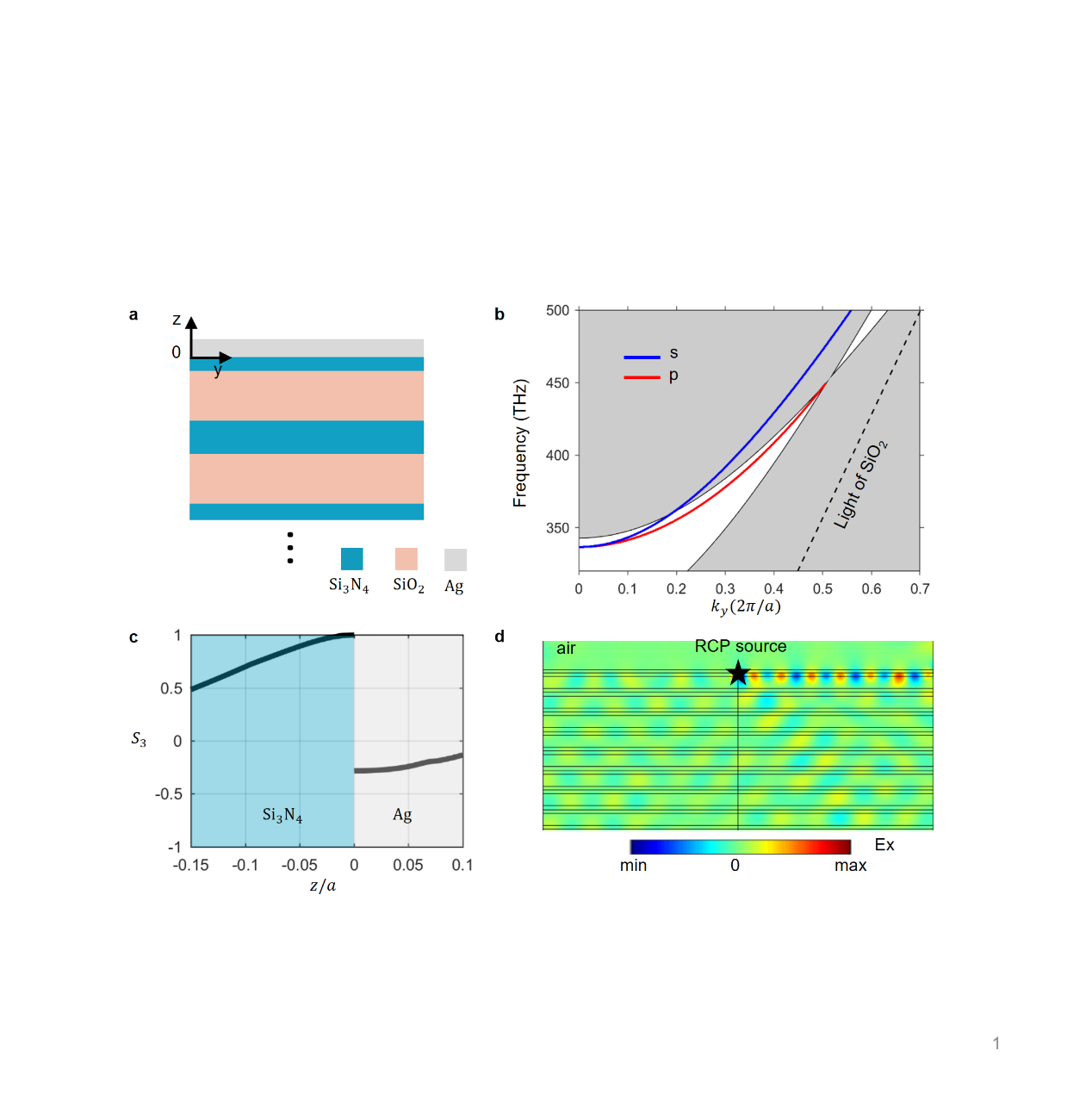


Fig. S8 (a) Schematic of the surface structure. (b) Projected band structure with s- (blue line) and p- (red line) polarized surface states. (c) Distribution of *S_3_* near the interface for the p-polarized surface state at $k_{y}=0.3\times2\pi/a$. (d) Excitation of p-polarized drumhead surface state by a right-handed circular-polarized source, which is indicated by the black star.

## 9. Propagation of the surface state at RPC_1_-RPC_2_ domain wall


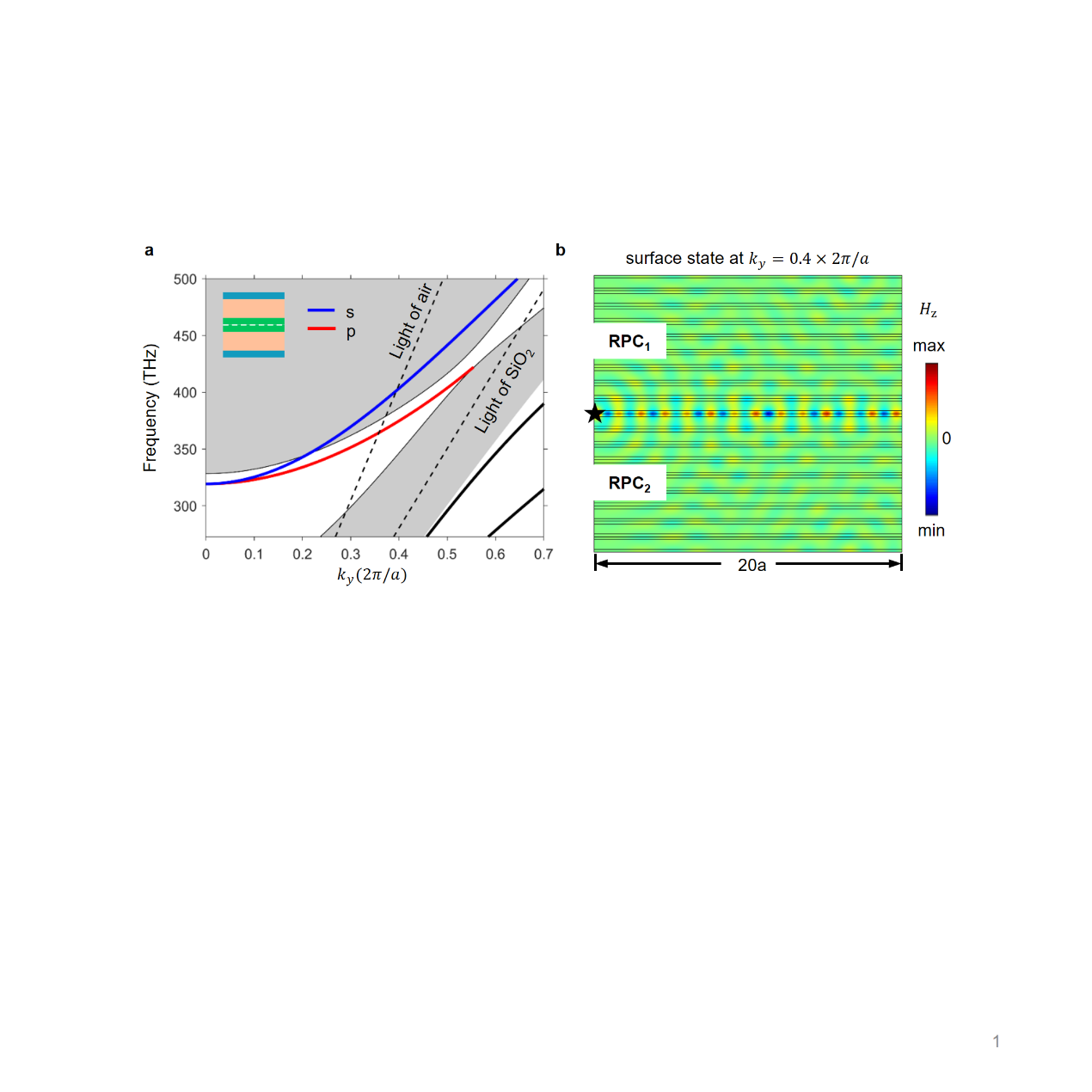


Fig. S9 (a) Projected band of RPC_1_-RPC_2_ domain wall. (b) Excitation of p-polarized surface state with $k_{y}=0.4\times2\pi/a$. The black star indicates the source’s position.

## 10. Estimation of the divergence angle induced by the focusing effect of cylindrical lens

Fig. S10 illustrates the focusing effect of cylindrical lens. The focus length is approximately $f=2Rn_{2}=23.2 mm$. Then, one can obtain $\sin\left( \theta\right)\approx r_{1}/f\approx0.02$, where $\theta$ is divergence angle and $r_{1}$ is the radius of the incident beam. The wavelength near the bulk nodal ring is $\lambda_{0}\approx650 nm$. Corresponding wavevector is $k_{0}=n_{2}\times2\pi/\lambda_{0}$. The lateral wavevector induced by the beam divergence is $\Delta k_{y}=k_{0}sin\theta\approx2.80\times{10}^{5} m^{-1}$, which is much smaller than the $k_{y}$ value at the nodal ring ($k_{y0}=0.513\times2\pi/a\approx1.11\times{10}^{7} m^{-1}$). So, the divergence of incident beam does not affect the observation of the nodal ring in the experiment.


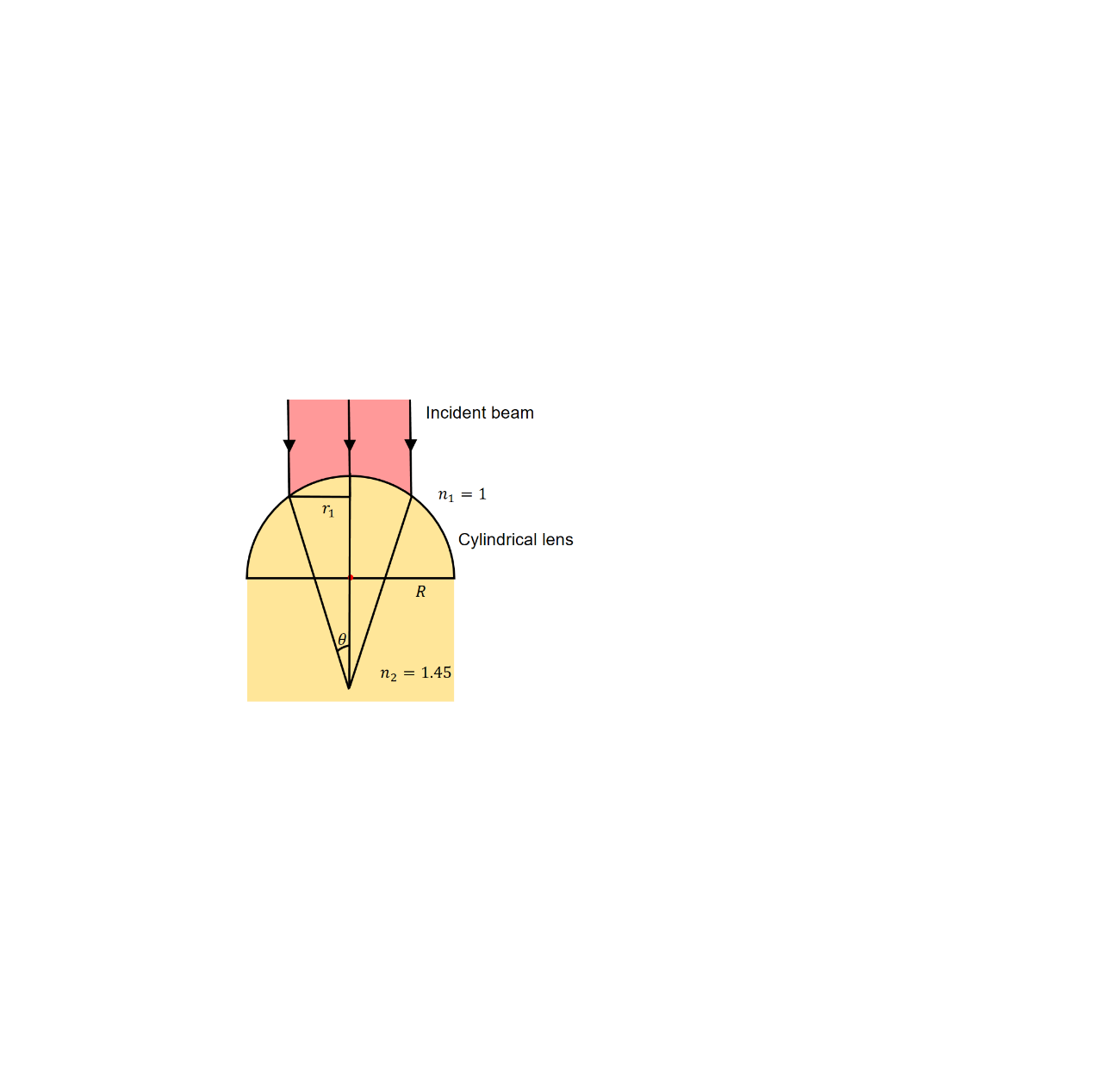


Fig. S10 Schematic of cylindrical lens’s focusing effect. The radius of the incident beam and cylindrical lens is $r_{1}=0.5 mm$ and $R=8 mm$, respectively.

**References**

1 Yariv, A. & Yeh, P. *Optical waves in crystals : propagation and control of laser radiation*. Wiley, New York (2003).

2 Aiello, A. et al,. From transverse angular momentum to photonic wheels. *Nature Photonics* **9**, 789-795 (2015).

3 Deng, W.-M. et al,. Transverse angular momentum in topological photonic crystals. *Journal of Optics* **20**, 014006-014013 (2018).

4 Ruan, W. S. et al,. Analysis of Unidirectional Coupling in Topological Valley Photonic Crystal Waveguides. *Journal of Lightwave Technology* **39**, 889-895 (2021).

5 Born, M. & Wolf, E. *Principles of optics: electromagnetic theory of propagation, interference and diffraction of light*. CUP Archive (2000).

6 Barik, S. et al,. A topological quantum optics interface. *Science* **359**, 666-668 (2018).
